# Nævoid Lipoma and Its Association with Hypertrophy of the Toes

**Published:** 1891-09-05

**Authors:** 


### NÆVOID LIPOMA, AND ITS ASSOCIATION WITH HYPERTROPHY OF THE TOES.

Nævoid lipoma is a comparatively rare growth. It is not described at any length in text books, but is very interesting both from a clinical and pathological point of view.

It must not be confused with fatty degeneration of a nævus. In this condition the nævus diminishes in size, there is a fatty degeneration, and the vascular spaces become filled with clot. In a nævoid lipoma the tumour is composed of a mixture of fatty and nævoid growth, and the fatty growth is very largely fibrous. It is, indeed, analogous to the common parotid tumour in which we have a mixture of gland tissue, cartilage, and myxomatous tissue.

It has been suggested that this is not the true view as to their pathology, but that they are merely fatty tumours with unusually large veins; but we have seen a case in which a subcutaneous nævoid lipoma was associated with a small undoubted capillary nævus of the skin over it, and in many of these tumours the venous spaces are much dilated so as to form in some parts blood cysts.

Again it has been suggested that the great vascularity has caused an increased growth of fat around the nævoid tissue, just as some observers have maintained that the hyperæmia in nut-meg liver causes fatty changes in the cells in the periphery of the lobules; but we cannot accept this theory, for, if correct, fatty tissues ought to be present in all nævi, and we must remember that the formation of fatty tissues is not the same thing as fatty degeneration. Congenital fatty tumours do sometimes contain cartilage and bone, why not then nævoid tissue occasionally?

Clinically, these growths appear like ordinary fatty tumours, and do not present any signs of undue vascularity. In the case to which we have already referred, this, however, was not the case; there was a capillary nævus of the skin, and a certain venous fulness around, apparently subcutaneous, but associated with this growth was another of the same nature, which presented no signs of venous fulness whatever. Ordinary fatty tumours, however, are rarely congenital. These growths usually are. They occupy the common position of fatty tumours, the back and nates, and have also been found on the lower limbs. Erichsen says they are only found on the back, nates, and thigh, but we have seen them (verified by removal) on the leg, and Butlin mentions two cases where they were present under the muscles over the ribs, and one where a growth of this nature was diffused amongst the structures of the sole of the foot.

Erichsen also says that in one case he operated on, the growth recurred three times after removal. But it was situated on the buttock, extending towards the perineum, and one cannot help thinking some corner or lobule may have been left behind, rather than such simple tumour elements should have shown such a character of malignancy.

We have seen these nævoid lipomata associated with congenital hypertrophy of the great toe in the same leg. This is very interesting when we remember that in "giant foot" the increased bulk is sometimes mainly due to excess of fatty tissue, and in others to distinct fatty growths associated with hypertrophy of all the tissues. Busch describes the case of a young man who had hypertrophy of the toes and irregular fatty growths on the foot also; and Butlin mentions a case in which marked hypertrophy of the great toe was found mainly due to excessive growth of fat, a condition to which our own case was probably analogous.

Jacobi attributes this congenital hypertrophy to intra-uterine venous congestion in early fœtal life; but nævoid lipoma can hardly be supposed to originate in this way, and the combination of definite tumours with the hypertrophy seems to me rather to point to a tendency to excessive development, such as we see in the breast sometimes at puberty, associated with another form of excessive tissue development, *i.e.*, the growth of definite tumours.

